# Release Kinetics Studies of Early-Stage Volatile Secondary Oxidation Products of Rapeseed Oil Emitted during the Deep-Frying Process

**DOI:** 10.3390/molecules26041006

**Published:** 2021-02-14

**Authors:** Tomasz Majchrzak, Andrzej Wasik

**Affiliations:** Department of Analytical Chemistry, Faculty of Chemistry, Gdańsk University of Technology, 80-233 Gdańsk, Poland; andrzej.wasik@pg.edu.pl

**Keywords:** deep-frying, volatiles, oil degradation products, food processing, proton transfer reaction mass spectrometry

## Abstract

The research concerns the use of proton transfer reaction mass spectrometer to track real-time emissions of volatile secondary oxidation products released from rapeseed oil as a result of deep-frying of potato cubes. Therefore, it was possible to observe a sudden increase of volatile organic compound (VOC) emissions caused by immersion of the food, accompanied by a sudden release of steam from a potato cube and a decrease of the oil temperature by more than 20 °C. It was possible to identify and monitor the emission of major secondary oxidation products such as saturated and unsaturated aldehydes, namely acrolein, pentanal, 2-hexenal, hexanal, 2-nonenal and 2-decenal. Each of them has an individual release characteristic. Moreover, the impact of different initial frying temperatures on release kinetics was investigated. Subsequently, it was possible to approximate the cumulative emission by a second-degree polynomial (R^2^ ≥ 0.994). Using the proposed solution made it possible for the first time to observe the impact of the immersion of food in vegetable oil on the early emission of thermal degradation products oil.

## 1. Introduction

As a result of thermal treatment, food becomes more easily digestible, microbiologically safe and gains desirable taste and aroma. Frying, one of the most commonly used methods of food thermal processing, is characterized by high process temperature and versatile application. As a result of frying the food acquires a characteristic texture, colour and palatability [1]. Moreover, the properties of the fried product depend, among other things, on the type and amount of oil used, frying time and temperature, and the cooking utensil in which the process is carried out [2].

Therefore, the control and modification of the frying process require the understanding of the detailed chemistry and of the physical processes that take place during frying. Therefore, studies are conducted on the texture of fried food [3] or identification of compounds responsible for food taste and flavour [4]. This is a consequence of multiple reactions taking place in food, oil, or between food and oil, including oxidation and hydrolysis of oil [2,5] or Maillard reaction [6,7]. In particular, the oxidation of fatty acids leading to secondary oxidation products (SOPs) can directly describe the frying behaviour, together with the loss of oil quality [8].

SOPs are mainly unsaturated and saturated aldehydes, furans, carboxylic acids and alcohols, the presence of which in oil fumes as well as the pathway of their formation is well known [9]. However, the studies to date have focused on long frying periods, i.e., over several hours [2]. It is very interesting to record how these compounds are released during the initial frying time, i.e., a few minutes or even seconds after the food is placed in the oil. Therefore, the knowledge gained in such a way could be used for example to modify the frying process in order to obtain food with desired taste qualities which might be highly relevant to the food processing industry. Due to the widespread use of gas chromatography in oil volatile degradation products analysis [10], often due to the necessity to take an oil sample and enrich volatile compounds, it is not possible to observe the changes of early forming volatile products. The most frequently used procedures of volatile fraction analysis include the use of static headspace (SHS) [11], dynamic headspace sampling (DHS) [12] and solid-phase microextraction (SPME) [13] coupled with gas chromatography. To enable true real-time measurement, novel, direct infusion mass spectrometers (DI-MS) can be applied.

An example of DI-MS that can be used to evaluate release kinetics of SOPs emitted during frying is proton transfer mass spectrometry (PTR-MS). This is a technique using soft chemical ionization, i.e., the reaction between the hydronium ion and the volatile compound [14]. Thus, it is possible to directly link the mass spectrum peak with a chemical compound found in oil fumes. PTR-MS is commonly used in food analysis [15,16,17], including oil quality analysis [18,19,20,21,22,23]. However, until now, this technique had limited application in this field, and was used e.g., in continuous measurement of oil fumes generated during frying [8] over a long period, lasting three hours, not focusing on early-stage SOPs release kinetics.

The research described here aimed at understanding the kinetics of volatile organic compound emissions, including SOPs, produced during deep-frying. To date, due to equipment limitations, it has not been possible to track the emission of these compounds second by second, from the moment of food immersion in hot oil to obtaining of the final product. It was made possible by using PTR-MS. The experiment was carried out for a model system using rapeseed oil and potato cubes, and the processing chamber was specially designed to ensure that the frying conditions were as constant as possible.

## 2. Results and Discussion

Despite the exclusion of many variables and measuring in fixed conditions, the oxidation of oil during deep-frying took place with different intensities, resulting in large discrepancies between repetitions. However, the way the recorded signal changed was very similar, so it was decided to normalize the signal in most cases. Additionally, to reduce the potential impact of water vapour on measurements, all data were normalized in relation to the H_3_O^+^ ion and expressed as normalized counts per second (ncps). Additionally, to show how the total volatile organic compouds (VOCs) flux changes, a total parameter (total volatile organic compounds–TVOC) has been introduced as the sum of ncps for VOCs for which the threshold signal was three times higher than the noise.

### 2.1. VOCs Emissions during Deep-Frying

**Figure 1:**
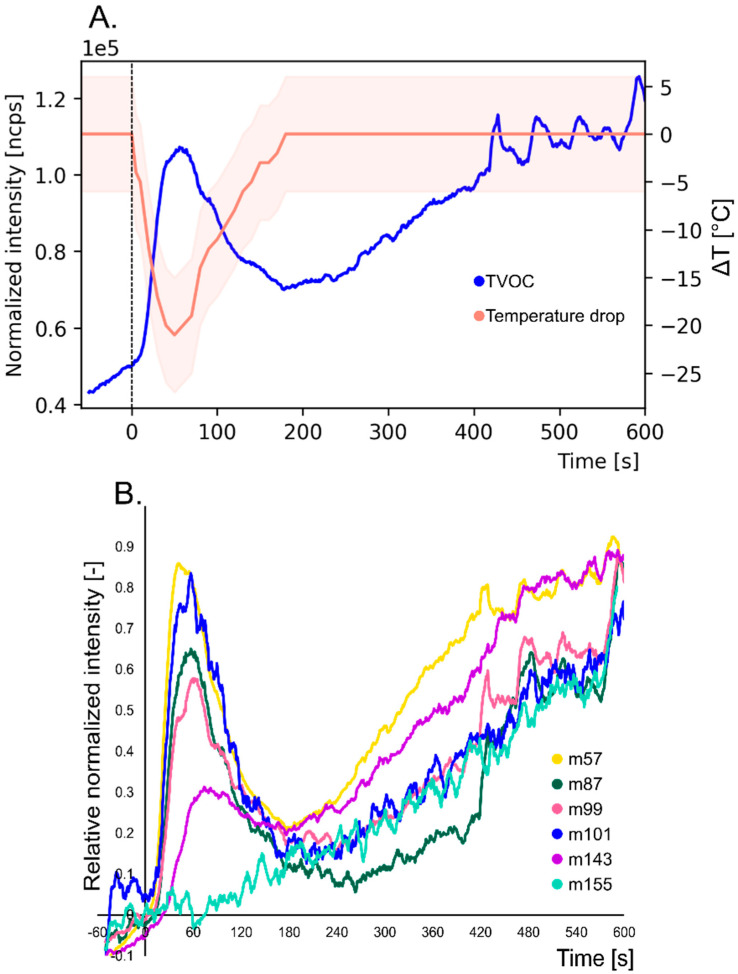
Emission profile of volatiles generated during potato cube deep-frying in rapeseed oil heated to 180 °C; (**A**) TVOC (total volatile organic compounds) changes during frying expressed as ncps and oil temperature drop during frying; (**B**) Selected ions, namely m57 (57.03 *m*/*z*), m87 (87.08 *m*/*z*), m99 (99.07 *m*/*z*), m101 (101.10 *m*/*z*), m143 (143.14 *m*/*z*), m155 (155.14 *m*/*z*) which represent SOPs (secondary oxidation products) emitted during deep-frying; start of frying at time 0 s.

Figure 1A shows the change in TVOC during deep frying of a potato cube in rapeseed oil heated to 180 °C. The total load of volatile products increases as the frying time progresses. In the diagram, time of 0 s determines when the potato was placed in the hot oil. A rather pronounced, sudden change at the beginning of the frying period is caused by the rapid formation of water vapour, which releases volatile degradation products from fried food and oil. The dynamic process of water vapour release can be shown by measuring the oil temperature. Characteristically, when the food is placed in the oil, the temperature drops drastically to reach a local minimum after one minute of frying - even beyond 20 °C of temperature decrease. After a minute of frying, it is also possible to measure the burst of volatiles, manifested as the high value of TVOC. In the next stage, the temperature returns to a constant 180 °C and the amount of produced VOCs is characterized by a linear increase. At this stage, a crust has already formed on the surface of the potato, which effectively stops water from getting out of the food bulk. The last minutes represent a situation in which too long a frying can lead to overcooking of the potato. The crust becomes darker, firmer and begins to crack. Thus, the graph depicts an accelerated fluctuation, as well as a gradual flattening of the TVOC curve. This full picture of TVOC emission during frying might not be possible to record in a situation of non-continuous measurement.

Considering the changes in the amount of selected chemical compounds produced during frying, the diversity of their emission profile can be observed. Figure 1B shows the emission profile of selected ions, namely m57 (57.03 *m/z*), m87 (87.08 *m/z*), m99 (99.07 *m/z*), m101 (101.10 *m/z*), m143 (143.14 *m/z*), m155 (155.14 *m/z*). These ions represent SOPs formed during the oxidation of rapeseed oil and may reflect the presence of: m57—acrolein (C_3_H_4_OH^+^), m87—pentanal (C_5_H_10_OH^+^), m99—2-hexenal (C_6_H_10_OH^+^), m101—hexanal (C_6_H_12_OH^+^), m143—2-nonanal (C_9_H_18_OH^+^), m155—2-decenal (C_10_H_18_OH^+^). Saturated and unsaturated aldehydes are recognized as the main SOPs formed during frying. However, the vast majority of studies were focused on investigating the emission during prolonged frying (more than 1 h) [24], lacking emphasis on early-stage release kinetics. The manner of emission of volatile compounds is presented as a relative signal in which the moment of placing a potato in oil assumes a zero value and the maximum recorded value is one. Thus, it was possible to depict all ions in the graph, regardless of their intensity. It can be seen that, similarly to the TVOC parameter, the ions m75, m87, m99, m101 and m143 are characterized by an initial sudden increase in the signal, reaching peak value and then returning to linear increase. The m155, on the other hand, is characterized by a linear increase throughout the whole measurement period. This phenomenon was possible to observe in other studies, where one group of aldehydes (including acrolein and pentanal) were characterized by higher concentrations in the early phase of frying while other aldehydes, like 2-decenal, have more flattened characteristics [2]. However, it should be noted that sampling was done with a lower frequency and the measurement itself did not concern oil fumes.

Moreover, a mass-dependent trend in bursting peak relative normalized intensity can be observed—the smaller the molecular weight, the higher the peak is. The graph also shows that with the increase of the molecular weight the bursting peak is delayed. This may be due to the general trend of decreasing volatility and increasing hydrophobicity as the carbon chain extends—sticky compounds may adsorb on the transmission line internal walls. 

### 2.2. The Role of Food in the Release Kinetics of SOPs

**Figure 2:**
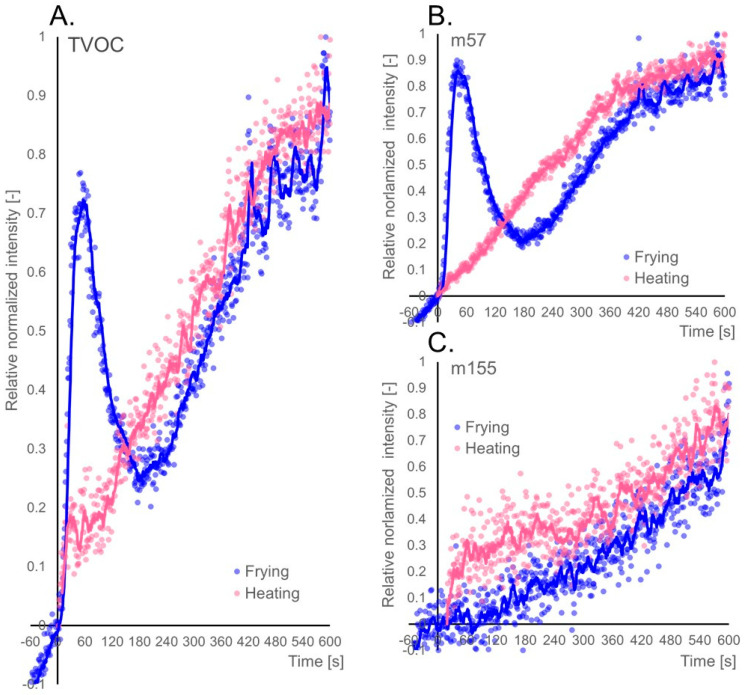
Comparison of the volatiles emission profile generated during potato cube deep-frying (blue) and rapeseed oil heating (pink) at 180 °C; Emission profiles of: (**A**)—TVOC (total volatile organic compounds) parameter; (**B**)—m57 ion, and (**C**)—m155 ion; start of frying at time 0 s.

To illustrate the effect of water vapour on the initial stage of TVOC emission, Figure 2A shows a normalized TVOC curve for oil heated at 180 °C (pink) and frying at the same temperature (blue). The main change in the TVOC emission pattern was the occurrence of the bursting peak. Besides, a distinct valley can be observed just after the bursting peak. This local lowering of the signal is much more evident in the case of m57 (Figure 2B), where the signal of about 200 s is lower for frying than for heating. This may be due to a temporary temperature drop caused by the potato cube being placed in the oil, and the subsequent delay in heating of oil, resulting in a less intensive oil oxidation process. At the end of the frying process, the emission of SOPs is similar in both cases. Therefore, a significant influence of food on the profile of SOPs emission during frying is in its early stage. In the case of other compounds, for example, for 2-decenal (m155) the emission is more dependent on the thermal degradation of oil than on the presence of food (Figure 2C). Naturally, it should be emphasized that the signal intensity during frying is much higher than in the case of heating alone. Therefore, it can be assumed that food with its developed, porous surface, when placed in oil allows the reaction to take place on its surface which may result in an overall increase of the signal.

Table 1 lists ncps for selected signals in the first, fifth and tenth minute of the experiment. It can be observed that compounds with a higher molecular weight are emitted in smaller amounts, which may be due to their lower volatility. A similar trend can be observed with the appearance of bursting peak in the first seconds of frying. For the m101, m143 and m155 peaks a gradual increase in signal intensity can be observed, and for the remaining peaks, a higher signal can be seen in the first minute of frying.

### 2.3. Effect of Temperature on the SOPs Emission

**Figure 3:**
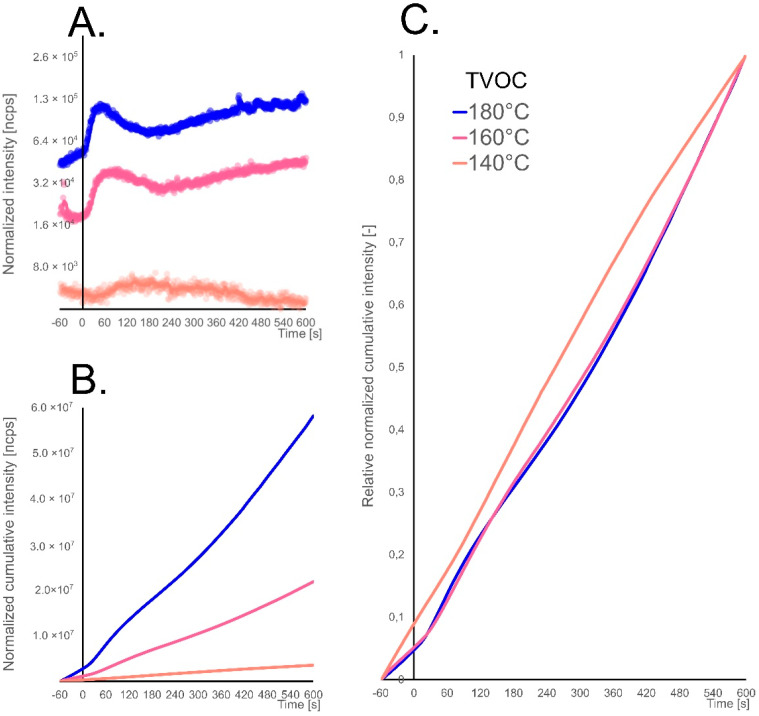
Comparison of emission profile (**A**), cumulative emission (**B**) and relative cumulative emission (**C**) expressed as TVOC (total volatile organic compounds) parameter during potato cube deep-frying in rapeseed oil for different frying temperatures, namely 140 °C, 160 °C and 180 °C; Start of frying at time 0 s.

A change in the frying temperature influences the frying process, which, in the context of food preparation, results in not obtaining the desired taste, improper texture or insufficient processing (in case of too low temperature). The next experiment aimed to check whether the nature of SOPs emission changes with the change of frying temperature. Figure 3A shows ncps signal of TVOC for 140 °C, 160 °C and 180 °C. As the first observation, the total signal intensity for TVOC increases with the frying temperature. Besides, it can be seen that the bursting peak at 180 °C is much higher and narrower than when frying at 160 °C, and when frying at 140 °C it does not occur. This is probably due to the faster evaporation of the water and the rapid formation of the crust on the potato cube in higher temperature. In the case of the lowest frying temperature, such a crust did not form. Also, a decreasing slope of SOPs emissions can be observed at a late stage of frying as its temperature drops. When frying at 140 °C, the TVOC parameter fluctuates around a constant value throughout whole experiment.

To examine the total amount of emitted compounds during the whole frying process, cumulative release curves were plotted (Figure 3B). This approach is applicable in tracking release kinetics, for instance in roasted coffee aroma determination [25]. This made it possible to record the total load of the arising compounds, and thus the total degree of oil degradation. This type of information may indicate the degree of oil oxidation. It can be observed that as the frying temperature increases, the oxidation occurs more dynamically. To describe the nature of the transformations taking place in the oil, the regression model was matched to the cumulative release curves. In the first step, relative cumulative release curves were prepared (Figure 3C). This gives a more universal character to release curves.

Table 2 shows the parameters of the regression model, which is a second-degree polynomial. Describing the curves with a quadratic function, the R^2^ match was obtained with a minimum of 0.999 for all TVOC release curves. Moreover, the polynomial parameters for selected ions were determined. For some of them, R^2^ was lower than the assumed 0.999, mainly in the case of 180 °C frying, which was caused by a greater dynamism of transformation, which is associated with the occurrence of local episodes of increased emission. However, also in the case of these compounds, a match not lower than R^2^ = 0.994 was obtained.

Analyzing the obtained release quadratic function curves equations it is possible to observe that most of the SOPs have similar emission pattern at the same frying temperature. It can be seen that as the frying temperature increases, parameters a and c increase, whereas b decreases. These changes have mostly linear characteristics.

## 3. Materials and Methods

### 3.1. Experimental Setup

**Figure 4:**
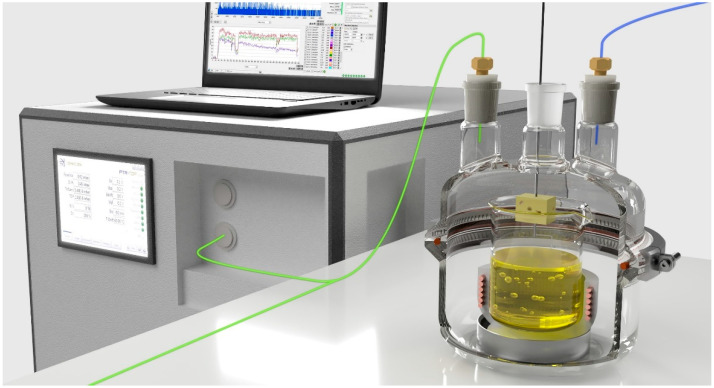
Experimental setup for early-stage SOPs (secondary oxidation products) monitoring.

The release kinetics of secondary emission products of rapeseed oil oxidation were measured using a special measuring setup, the visualization of which is shown in Figure 4. Clean air with the flow rate of 500 sccm was delivered to a glass chamber, in which a heating jacket with a 50 mL beaker with rapeseed oil was placed. The heating was controlled by a PID regulator equipped with a K-type thermocouple (Labafacility Ltd., Bognor Regis, UK). This made it possible to maintain oil temperature during frying with an accuracy of ± 6.0 °C. Oil fumes were transported in 1/8 in. PTFE tubing outside the reaction chamber. The transmission line was equipped with a filter to stop the oil droplets and condensate. Connected with the use of T-piece heated to 70 °C 1/16 in PEEK capillary transported oil fumes with flow rate of 100 sccm to the PTR-MS instrument (Ionicon GmbH, Innsbruck, Austria). The remaining oil fumes were exhausted from the system. Before the oil fumes entered the PTR-MS system they were diluted 1000-times with dry clean air heated to 70 °C. This made it possible to avoid overloading of the mass spectrometer as well as to reduce the humidity of the gas stream entering the PTR-MS. Excessive humidity could lead to the formation of a large number of clusters (H_2_O)_n_H_3_O^+^ [26]. This in turn could lead to the appearance of a significant amount of ions resulting from the proton exchange reaction with water clusters instead of protonated parent ions [27].

The frying was conducted for 10 min, during which the MS spectrum was recorded every second. The experiment was carried out at 180 °C, which is a typical frying temperature [28], and at lower temperatures, that is 160 °C and 140 °C. After frying, the potato cube was immediately removed from the oil, the system was opened and a sample of the oil was taken to assess its quality. Measurements using PTR-MS were carried out in triplicate (Figure S1; Supplementary Materials).

### 3.2. Materials

In this experiment, potato cubes were fried in rapeseed oil. Throughout the experiment, one variety of potato was used, namely the Polish Gala variety, which is culinary type B (general use). The choice of potato was mainly due to its popularity and widespread use, as well as for practical reasons-it enabled producing cubes for frying with a consistent size. The potato was cut into a regular cube of 1.5 cm × 1.5 cm (±0.2 cm) and weight of 5 g (±0.1 g). The potato cube contained about 76.2 ± 2.6% of water (loss on drying method). Oil from the same manufacturer and the same batch of oil (same best before date) was used in the tests. The oil, both before and after the experiment, was tested for thermal stability using the accelerated oxidation method Rancimat (procedure described in [22]). Thus, it was possible to indicate that the thermal stability time was reduced by 26.4 ± 3.2% (from approx. 5.8 to 4.3 h).

### 3.3. PTR-MS

The PTR TOF1000 ultra proton transfer reaction mass spectrometer (Ionicon GmbH) was used in this research. A reduced field E/N of 100 Td was maintained during the measurement. The 1000 sccm of diluted sample was transferred to the system, the drift chamber was kept at 70 °C. During the measurement, internal two-point *m*/*z* calibration (NO^+^ and 1.3-diiodobenzene fragment) was carried out due to the use of PerMaSCal permeation source. The IoniTOF v3.0 (Ionicon GmbH) and PTR-MS Viewer v3.3.7 (Ionicon GmbH) software were used for data acquisition and processing, respectively. Data processing was carried out using a customized script in R.

## 4. Conclusions

Using PTR-MS it was possible to trace the release characteristic SOPs of rapeseed oil during the frying process. It was possible to observe that the volatile oil oxidation products burst in the first seconds after placing food in hot oil. Thus, the emission is influenced by the food’s changes such as the release of a large amount of steam as well as the formation of crust on the surface of the fried food. The SOPs emission is strongly influenced by the frying temperature. In the research, it was possible to describe the characteristics of SOPs emission based on the quadratic function.

Until now, due to the lack of suitable tools, it has not been possible to determine the exact changes that occur in the early stages of frying. By gaining this knowledge, it is possible to better understand the process and to determine the role of frying parameters not only on the taste of food but also on the safety of consumers or workers in the catering.industry exposed to SOPs. Moreover, knowledge of the kinetics of SOPs release can be used to modify the frying process in terms of appropriate taste or oil quality.

## Figures and Tables

**Table 1:** Intensity (ncps) of selected SOPs (secondary oxidation products) of rapeseed oil in the first, fifth and tenth minute of frying.

Peak Signature	Molecular Formula	m/z	Tentatively Identified Compound	Normalized Peak Intensity [ncps] ^a^		
				1 min	5 min	10 min
m57	C_3_H_4_OH^+^	57.03	Acrolein	3.14 ± 0.22 × 10^4^	2.51 ± 0.14 × 10^4^	3.33 ± 0.13 × 10^4^
m87	C_5_H_10_OH^+^	87.08	Pentanal	5.31 ± 0.60 × 10^3^	4.56 ± 0.44 × 10^3^	4.67 ± 0.47 × 10^3^
m99	C_6_H_10_OH^+^	99.08	2-Hexenal	3.76 ± 0.46 × 10^3^	3.52 ± 0.44 × 10^3^	4.10 ± 0.45 × 10^3^
m101	C_6_H_12_OH^+^	101.10	Hexanal	2.39 ± 0.37 × 10^3^	2.45 ± 0.17 × 10^3^	2.50 ± 0.26 × 10^3^
m143	C_9_H_18_OH^+^	143.14	2-Nonenal	2.62 ± 0.48 × 10^3^	4.44 ± 0.57 × 10^3^	5.10 ± 0.53 × 10^3^
m155	C_10_H_18_OH^+^	155.14	2-Decenal	0.51 ± 0.11 × 10^3^	1.12 ± 0.13 × 10^3^	1.23 ± 0.18 × 10^3^

^a^ signal expressed as the average value from 10 s of measurement ± SD.

**Table 2:** Quadratic function parameters describing cumulative release kinetics curves of rapeseed oil SOPs (secondary oxidation products).

Parameter	*ncps_cum_= a (time)^2^ + b(time) + c*		
	140 °C	160 °C	180 °C
**TVOC**			
**a [×10^−7^]**	−2.39	4.96	6.07
**b [×10^−3^]**	1.69	1.25	1.18
**c [×10^−2^]**	8.24	5.96	6.24
**m57**			
**a [×10^−7^]**	−8.76	0.10^a^	7.97 ^a^
**b [×10^−3^]**	2.00	0.95^a^	1.08 ^a^
**c [×10^−2^]**	0.12	4.10^a^	6.67 ^a^
**m87**			
**a [×10^−7^]**	−5.81	1.49	4.04 ^c^
**b [×10^−3^]**	1.82	1.42	1.22 ^c^
**c [×10^−2^]**	0.12	7.92	8.61 ^c^
**m99**			
**a [×10^−7^]**	−7.01	3.31	6.70 ^b^
**b [×10^−3^]**	1.90	1.31	1.10 ^b^
**c [×10^−2^]**	0.11	7.74	6.72 ^b^
**m101**			
**a [×10^−7^]**	−7.00	−2.53	1.02 ^d^
**b [×10^−3^]**	1.89	1.65	1.36 ^d^
**c [×10^−2^]**	0.12	9.24	10.8 ^d^
**m143**			
**a [×10^−7^]**	−2.44	7.18	13.0
**b [×10^−3^]**	1.66	1.13	0.79
**c [×10^−2^]**	0.10	6.20	4.51
**m155**			
**a [×10^−7^]**	−4.89	−0.01	6.97 ^a^
**b [×10^−3^]**	1.78	1.51	1.06 ^a^
**c [×10^−2^]**	0.11	8.97	8.55 ^a^

^a^ R^2^ = 0.998; ^b^ R^2^ = 0.997; ^c^ R^2^ = 0.995; ^d^ R^2^ = 0.994.

## Data Availability

The data presented in this study are available on the request from the corresponding author.

## Supplementary Materials

The following is available online. Figure S1. Graphical representation of three consecutive repetition emission profiles of m99 and m143 ion generated during potato cube deep-frying in rapeseed oil heated to 180 °C; start of frying at time 0 s.
