# Supplementary material for: Release Kinetics Studies of Early-Stage Volatile Secondary Oxidation Products of Rapeseed Oil Emitted during the Deep-Frying Process
Source: Molecules. 2021 Feb 14;26(4):1006. doi: 10.3390/molecules26041006 (PMC7918898; doi:10.3390/molecules26041006)
Supplement: Supplementary file 1 [file molecules-26-01006-s001.pdf]

Supplementary materials

Release kinetics studies of early-stage volatile secondary oxidation products of rapeseed oil emitted during the deep-frying process

Tomasz Majchrzak [1,*], Andrzej Wasik [1]

[1] Department of Analytical Chemistry, Faculty of Chemistry, Gdańsk University of Technology, Gdańsk, Poland; tomasz.majchrzak@pg.edu.pl, andrzej.wasik@pg.edu.pl
* Correspondence: tomasz.majchrzak@pg.edu.pl

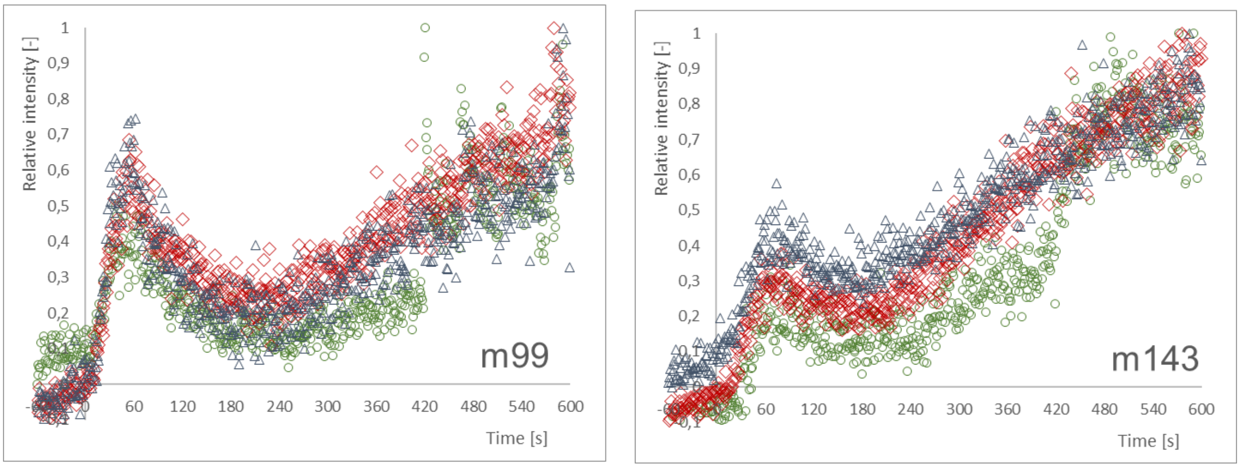

Figure S1. Graphical representation of three consecutive repetition emission profiles of m99 and m143 ion generated during potato cube deep-frying in rapeseed oil heated to 180°C; start of frying at time 0 seconds.
